# Neuroprotective Effects of Exogenous Activin A on Oxygen-Glucose Deprivation in PC12 Cells

**DOI:** 10.3390/molecules17010315

**Published:** 2011-12-30

**Authors:** Jin-Ting He, Jing Mang, Chun-Li Mei, Le Yang, Jiao-Qi Wang, Ying Xing, Hong Yang, Zhong-Xin Xu

**Affiliations:** 1 Department of Neurology, China-Japan Friendship Hospital, Jilin University, Changchun 130012, China; Email: Hejinting333@gmail.com (J.-T.H.); Mangjing505@sina.com (J.M.); wangjiaoqi111@sina.com (J.-Q.W.); xingying333@sina.com (Y.X.); yanghong555@sina.com (H.Y.); 2 College of Nursing, Beihua University, Jilin 132013, China; Email: meixiaoqing2007@126.com; 3 People’s Hospital of Jilin Province, Changchun 130021, China; Email:Yangle800511@sina.com

**Keywords:** OGD, tolerance model, exogenous activin A

## Abstract

Ischemic cerebrovascular disease is one of the most common causes of death in the World. Exogenous activin A (ActA) protects neurons against toxicity and plays a central role in regulating the brain’s response to injury. In the present study, we investigated the mechanisms involved in the neuroprotective effects of ActA in a model of hypoxic-ischemic brain disease. We found that ActA could effectively increase the survival rate of PC12 cells and relieve oxygen-glucose deprivation (OGD) damage. To clarify the neuroprotective mechanisms of ActA, the effects of ActA on the ActA/Smad pathway and on the up-regulation of inducible nitric oxide synthase (NOS) and superoxide dismutase (SOD) were investigated using OGD in PC12 cells. The results showed that ActA could increase the expression of activin receptor IIA (ActRIIA), Smad3 and Smad4 and that 50 ng/mL and 100 ng/mL of ActA could reduce NO levels and increase SOD activity by 78.9% and 79.9%, respectively. These results suggested that the neuroprotective effects of ActA in ischemia could be related to the activation of the ActA/Smad signaling pathway and to its anti-oxidant activities.

## 1. Introduction

Ischemic stroke occurs when the blood supply to the brain is obstructed, and it is one of the most common causes of health problems, disability and death in the World. Accumulating evidence suggests that the cell death observed during the first few hours of cerebellar ischemia is a result of apoptosis as opposed to necrosis, which was considered the predominant form of cerebellar damage generated by ischemia [1]. Moreover, the ischemic damage of nerve cells leads to the disruption of a series of complex signaling pathways that produces an effect on corresponding biological functions and affects the function of the brain; this terminal differentiated profile of the brain is of particular relevance for cerebellar ischemia [2,3,4]. Therefore, it is important to develop therapies that enhance the neuroprotective effects by inhibiting the mechanisms that lead to apoptosis and excessive cell death, before the process becomes irreversible.

Activins are the members of the transforming growth factor (TGF)-β superfamily, a group of multifunctional cytokines that regulate cell proliferation, differentiation and death [5]. These biologically active proteins are formed by the homo-or heterodimerization of two (activin) subunits to produce activin A (bA/bA), activin B (bB/bB) or activin AB (bA/bB) [6]. As multifunctional growth factors, the roles of the activins extend beyond the scope of the endocrine system and include the control of growth, development, immune function and other cellular processes [7]. Recent reports revealed a crucial role for ActA in inflammatory and repair processes involved in wound healing and brain injury [8]. ActA plays a central role in regulating the brain’s response to injury and has a significant protective effects [9,10].

The PC12 cell line is widely used as a model for dopaminergic neuronsm, because it possesses intracellular substrates for the synthesis, metabolism and transportation of dopamine (DA) [11]. An OGD-damage model is one of the more commonly used models for the study of cerebral ischemia. The principle of the OGD model is that Na_2_S_2_O_4_ quickly clears the oxygen in the culture matrix, does not damage the cell membrane and is better able to simulate the hypoxic environment when compared to the *in vivo* model [12]. In this study, the OGD of PC12 cells was used to establish a cerebral hypoxia-ischemia model. In addition, we measured the amount of NO and the induction of SOD in an attempt to elucidate possible mechanisms that underlie ActA-mediated protection against OGD in PC12 cells.

## 2. Results

### 2.1. Effects of ActA on Cell Proliferation

**Figure 1:**
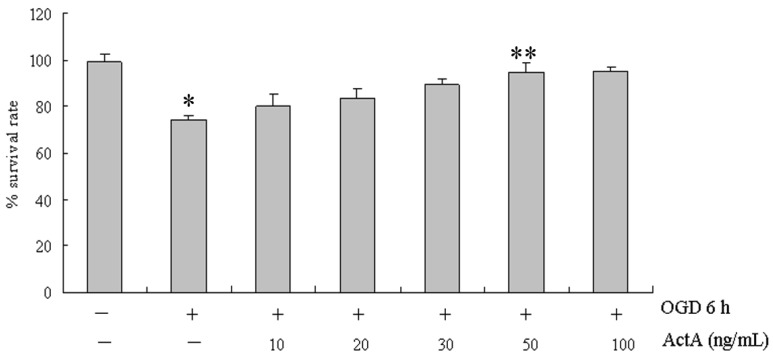
MTT assay showing the growth inhibition of PC12 cells treated with ActA for 24 h plus OGD for 6 h. The cells were grown to a density of 5 × 10^4^ cells per well in a 96-well plate for 24 h. The results show the growth of PC12 cells following incubation in 96-well plates for 24 h with 10, 20, 30, 50 and 100 ng/mL ActA plus OGD for 6 h, compared with OGD for 6 h. OGD 6 h/ActA^−^denotes a significant difference from the OGD^−^/ActA^−^group (*p* < 0.05); OGD 6 h/ActA 50 ng/mL denotes a significant difference from the OGD 6 h/ActA^−^group (*p* < 0.05). The data represent the means ± S.E.M. obtained from three separate experiments that were performed in triplicate.

The cytotoxicity of OGD for 6 h and ActA plus OGD for 6 h treatments were determined by examining their effects on the proliferation of PC12 cells. PC12 cells were treated with 10, 20, 30, 50 and 100 ng/mL ActA for 24 h before OGD for 6 h (Figure 1). MTT assays showed that treatment with OGD for 6 h effectively inhibited the growth of PC12 cells by 25.61%. Compared to the OGD for 6 h-treated group, the ActA plus OGD for 6 h-treated group inhibited the growth of PC12 cells by 19.82%, 16.31%, 10.36%, 5.41% and 4.92%, in dose-dependent manner, and the survival rates were higher than in the OGD for 6 h-treated group. Because there was no significant difference (*p* > 0.05) between the survival rates of the 50 and 100 ng/mL ActA-treated cells, 50 ng/mL ActA was used in all subsequent experiments.

### 2.2. ActA Anti-Apoptotic in PC12 Cells

**Figure 2:**
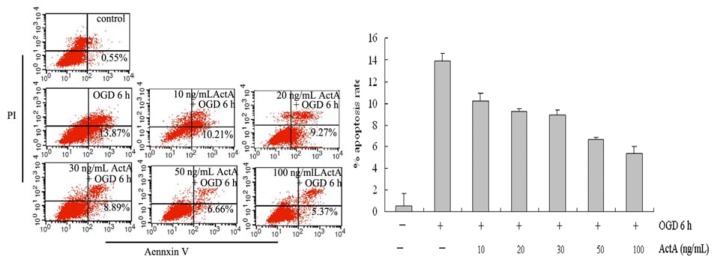
Flow cytometric analysis: assessment of apoptosis. OGD for 6 h treatment induced apoptosis in PC12 cells and ActA reduced cell damage. The cells were treated with OGD for 6 h or with different concentrations of ActA plus OGD for 6 h and were analyzed using flow cytometry following AnnexinV-FITC/PI staining. The data represent from three separate experiments that were performed in triplicate.

To assess the apoptosis of differentiated PC12 cells treated with ActA plus OGD and OGD alone, the cells were analyzed using Annexin V-FITC and PI double-staining flow cytometry. The signals from each group of cells are located in the lower right quadrant of the dot-plot graph, and the results are shown in Figure 2. Compared to OGD 6 h, the proportions of apoptotic cells treated with 10, 20, 30, 50 and 100 ng/mL ActA plus OGD for 6 h were 10.21%, 9.27%, 8.89%, 6.66% and, 5.37%, respectively. In the OGD 6 h group, the proportions of apoptotic cells was significantly higher than in the OGD^−^/ActA^−^ group; the apoptosis rate of the ActA plus OGD for 6 h-treated groups decreased significantly compared to OGD 6 h group, suggesting that ActA reduce cell damage. Compared with the 50 ng/mL ActA-treated group, the apoptosis rate of the 100 ng/mL ActA-treated group was slightly lower.

### 2.3. Caspase-3 Activation

**Figure 3:**
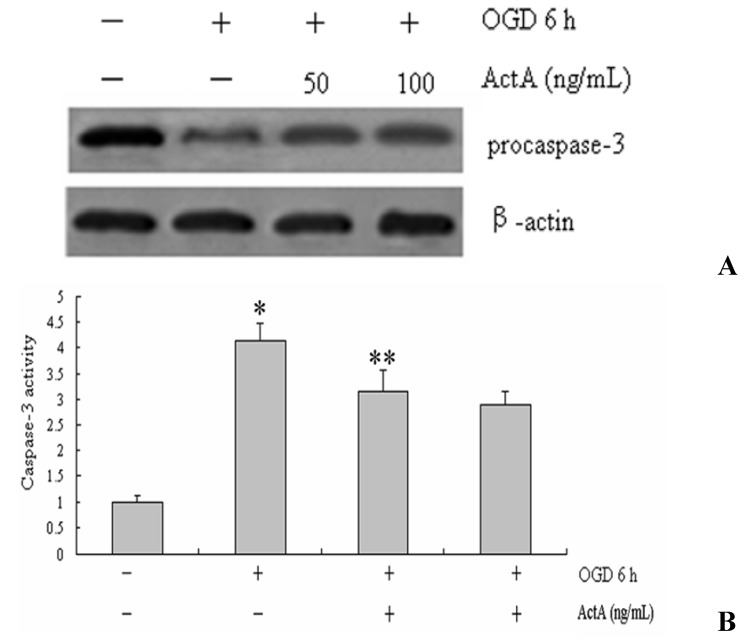
(**A**) Western blot analysis. PC12 cells were treated OGD 6 h and ActA for 24 h. Pro-capsase-3 expressions were determined by western blotting. The results are showed in representative of three repeated experiments. NIH imaging indicated that the protein signal densities were higher in OGD^+^/ActA^+^ group treated than in OGD^+^/ActA^−^group; (**B**) Effects of ActA on caspase-3 activity in PC12 cells. The cells were treated OGD 6 h and ActA for 24 h and analyzed for the activity of caspase-3. OGD 6 h/ActA^−^denotes a significant difference from the OGD^−^/ActA^−^ group (*p* < 0.05); OGD 6 h/ActA 50 ng/mL denotes a significant difference from the OGD 6 h/ActA^−^group (*p* < 0.05). The data represent from three separate experiments that were performed in triplicate.

Caspase-3 is widely activated in all major apoptotic signal transduction pathways, we tested pro-caspase-3 after OGD 6 h and ActA for 24 h. Compared with the OGD^−^/ActA^−^ group, the pro-caspase 3, expression levels in PC12 cells increased in the OGD for the 6 h-treated group. Compared with OGD 6 h, the expression levels of pro-caspase-3 increased in the groups treated with ActA for 24 h combined with OGD for 6 h (Figure 3A). To confirm the activity of caspase 3, we measured caspase-3 activities for OGD 6 h and ActA for 24 h. Compared with the OGD^+^/ActA^−^ group, the activities of caspase-3 decreased by treated with ActA for 24 h combined with OGD for 6 h (Figure 3B).

### 2.4. Effects of ActA on ActA/Smad Pathway

**Figure 4:**
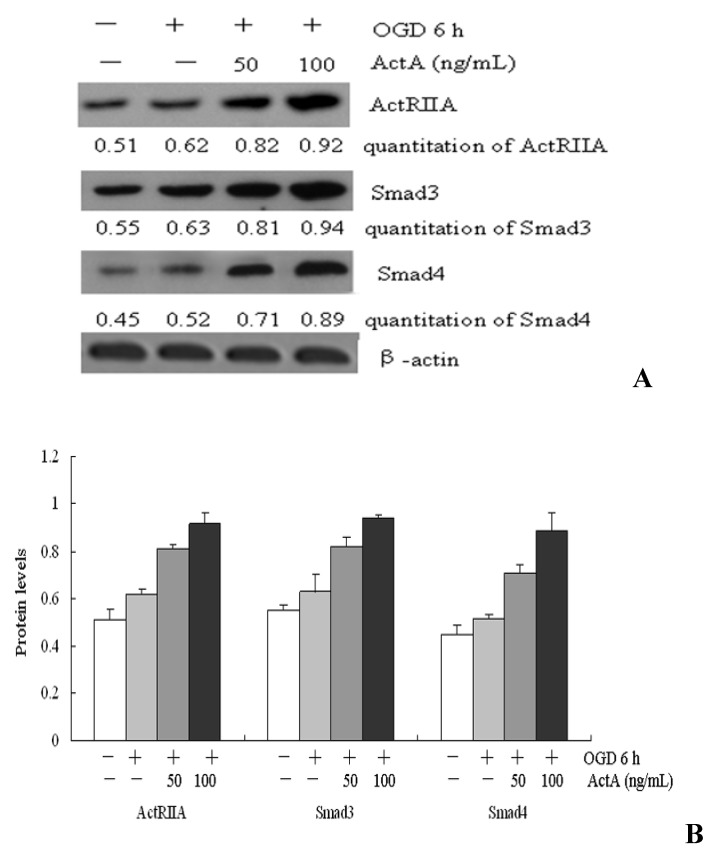
Western blot analysis. PC12 cells were treated with OGD for 6 h or different concentrations of ActA for 24 h combined with OGD for 6 h. (**A**) ActRIIA, Smad3 and Smad4 expression levels were determined using western blots. The results shown are representative of three independent experiments. NIH imaging indicated that the protein signal densities increased in the groups treated with ActA combined with OGD for 6 h compared with OGD for 6 h-treated groups; (**B**)ActRIIA, Smad3 and Smad4 protein levels inthe groups treated with ActA combined with OGD for 6 h compared with OGD for 6 h-treatedgroups. The data represent from three separate experiments that were performed in triplicate.

The ActA/Smad pathway plays a protective role in ischemia following its activation [13]. To investigate the neuroprotective mechanisms of ActA, the expression of ActRIIA, Smad3 and Smad4 were examined using Western blots (Figure 4). Compared with the OGD^−^/ActA^−^ group, the ActRIIA, Smad3 and Smad4 expression levels in PC12 cells increased in the OGD for the 6 h-treated group. Compared with OGD 6 h, the expression levels of ActRIIA, Smad3 and Smad4 increased in the groups treated with ActA for 24 h combined with OGD for 6 h. In this group, as the concentration of ActA increased, the expression levels of ActRIIA, Smad3 and Smad4 increased in a dose-dependent manner. Therefore, ActA may mediate the neuroprotective effect of the ActA/Smad signaling pathway.

### 2.5. Inhibition of NO and iNOS by ActA

**Figure 5:**
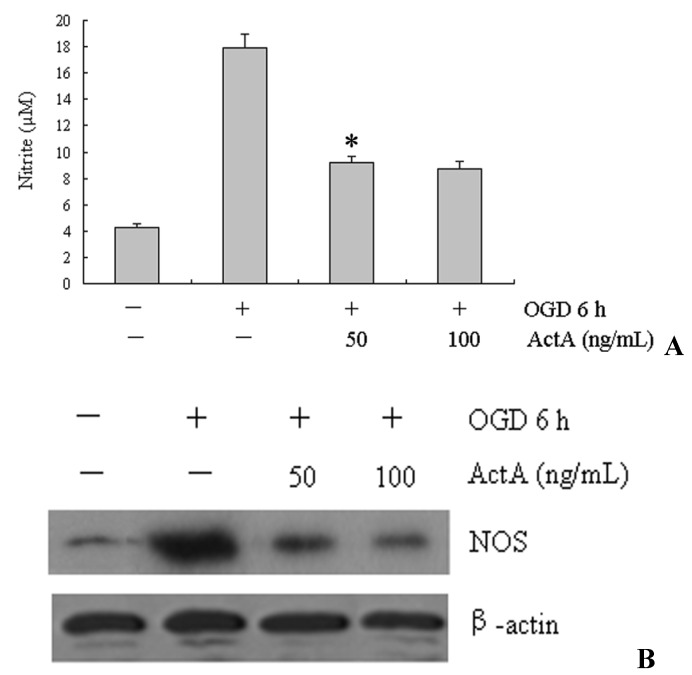
Effect of ActA on oxidative stress in OGD for 6 h-induced PC12 cells. (**A**) Cells were OGD-induced by incubation with 50 or 100 ng/mL of ActA for 24 h combined with OGD for 6 h. The nitrite content was measured using the Griess reaction. The values indicate the nitrite production in cells that were exposed to the culture supernatants collected from cells that were treated with OGD alone or cells that were exposed to OGD plus ActA. OGD 6 h/ActA 50 ng/mL denotes a significant difference from the OGD 6 h/ActA^−^ group (*p* < 0.05). The data represent the means ± S.E.M. from three independent experiments that were performed in triplicate; (**B**) Western blot analysis of OGD-induced cells or cells treated with increasing concentrations (50 and 100 ng/mL) of ActA for 24 h combined with OCD for 6 h. The western blot shows the levels of NOS protein expression. β-actin was used as an internal control. The data represent from three separate experiments that were performed in triplicate.

To understand further the neuroprotective mechanisms of ActA, we investigated if ActA could protect PC12 cells from oxidative injury. The cells were treated with OGD for 6 h or with 50 ng/mL ActA for 24 h plus OGD for 6 h, and NO production was assayed by measuring the levels of a stable NO metabolite, nitrites, in the conditioned medium. Incubation with ActA (50 ng/mL) for 24 h effectively inhibited NO production in OGD-stimulated PC12 cells (Figure 5AWestern blot analysis. As shown in Figure 5B, the treatment with 50 ng/mL ActA resulted in a significant decrease in NOS protein levels.

### 2.6. Increasing the Activity of SOD by ActA

**Figure 6:**
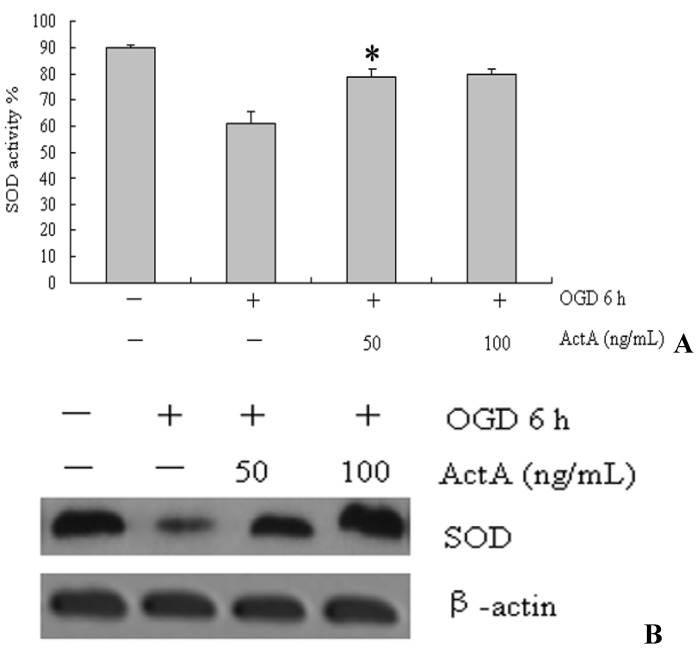
The effect of ActA on SOD activity. (**A**) Cells were OGD-induced or were treated with 50 or 100 ng/mL of ActA for 24 h plus OGD for 6 h. The effect of ActA on SOD activity was assessed. OGD 6 h/ActA 50 ng/mL denotes a significant difference from the OGD 6 h/ActA^−^ group (*p* < 0.05). The data represent the means ± S.E.M. obtained from three independent experiments that were performed in triplicate; (**B**) Western blot analysis of cells treated with OGD for 6 h or cells treated with OGD for 6 h in combination with increasing concentrations (50 and 100 ng/mL) of ActA for 24 h. The western blot shows the SOD protein expression. β-actin was used as an internal control.

The effect of ActA on SOD activity and protein expression were examined (Figure 6). As shown in Figure 6A, compared with the OGD^−^/ActA^−^ group, the SOD activity in PC12 cells decreased in the OGD for 6 h-treated group. Compared with the OGD for 6 h-treated group, the SOD activity increased following treatment with different concentrations of ActA for 24 h combined with OGD for 6 h. Furthermore, the treatment with 50 or 100 ng/mL ActA led to a significant increase in SOD protein levels (Figure 6B).

## 3. Discussion

Activin A, its receptors, and binding proteins are widely distributed throughout the brain [9]. The models of acute brain injury implicated enhanced activin A expression as a common response to acute neuronal damage of various origins [14]. Hypoxic/ischemic injury, mechanical irritation, and chemical damage of brain evoke a strong upregulation of activin A. Subsequent experimental studies have shown that activin A has a beneficial role to neuronal recovery and that, by activating different pathways, activin A has robust neuroprotective activities [15]. 

In this study, oxygen and glucose deprivation was applied to PC12 cells and the results show that the OGD for 6 h-treated group effectively inhibited the growth of PC12 cells, indicating that the cell model causes cell damage. When compared with the OGD for 6 h-treated group, the ActA plus OGD for 6 h-treated groups effectively increased the survival rate of PC12 cells (Figure 1, Figure 2 and Figure 3). Our results further confirmed the exogenous ActA neuroprotective effect on OGD-induced cell injury which might provide a new method of ischemic cerebrovascular disease treatment.

Activins are members of the transforming growth factor (TGF)-β superfamily, it regulated cellular growth and differentiation, control morphogenesis and angiogenesis and repair processes involved in wound healing and brain injury [8], it is possible that when the transmembrane activin receptor (ActR) combines with activin, which subsequently propagates the signal downstream by phosphorylating specific receptor-regulated Smad proteins (R-Smads). Phosphorylated R-Smads form heterocomplexes with the common partner Smad4 (Co-Smad) and translocate to the nucleus where they participate in the regulation of transcription of target genes [16,17] which in turn activates intracellular signal transduction pathways [18]. Smad2, Smad3, a total adjustment type Smad4 and inhibitory Smad7 participate in the ActA/Smad pathway. ActA may combine with ActRII to activate smad2 and smad3 and to promote the dimerization of smad4 and smad2/3, which enter the nucleus to influence gene transcription and the activation of a series of biological functions [19]. Our results demonstrate that treatment with OGD for 6 h increases the expression of ActRIIA, Smad3 and Smad4. When compared to the OGD for 6 h-treated group, ActRIIA, Smad3 and Smad4 expression levels increased following treatment with different concentrations of ActA for 24 h combined with treatment with OGD for 6 h (Figure 4). ActA may mediate the ActA/Smad signaling pathway to induce a neuroprotective effect.

The pathogenesis of ischemic cerebrovascular disease is complex and is related to electrolyte imbalances, the production of oxygen free radicals, lipid peroxidation, NO, monoamine neurotransmitters, phospholipid metabolism and the synthesis of other damaging factors [20]. The generation of oxygen-free radicals and NO plays an important role in the promotion of ischemic cerebrovascular disease [21]. Existing animal models are affected by many factors; therefore, a stable cell model of cerebral ischemia was used in this study because the ability to control the experimental conditions, the small amount of sample required and the short experimental period has clear advantages. Many studies indicate that oxidative stress plays a key role in ischemic cerebrovascular disease. SOD plays a vital role in the body’s oxidant and antioxidant balance by removing superoxide anion radicals and protecting cells from damage. NO is an important messenger and effector molecule *in vivo*, and plays a role in neurotransmitter functions in physiological and pathological events, therefore, SOD and NO are involved in oxidative damage. The results show that OGD significantly increases NO levels and decreases SOD activity, whereas ActA reduces NO levels and increases SOD activity, and this may be the mechanism by which ActA promotes the neuroprotective effect (Figure 5 and Figure 6). Additional *in vitro* and *in vivo* studies are necessary to determine whether ActA can be used as a preventive agent against neurodegenerative conditions, or to reduce the progression of chronic and neurodegenerative disorders.

## 4. Experimental

### 4.1. Cell Culture

PC12 cells were purchased from the Cell Bank of the Chinese Academy of Sciences. The cell line was maintained in DMEM medium supplemented with 10% (v/v) fetal bovine serum, 5% horse serum (FBS, GIBCO), 100 IU/mL streptomycin, 100 IU/mL penicillin, pH 7.0, and the cells were detached using 0.25% trypsin (Sigma, New York, NY, USA) [22,23]. PC12 cells were grown at 37 °C in 5% CO_2_.

### 4.2. OGD for in Vitro Ischemia

Before differentiation, the cells were grown in 5% horse serum-containing media on collagen-coated tissue culture dishes. After the cells were attached, they were treated with 100 ng/mL nerve growth factor (NGF 2.5S; Promega, Madison, WI, USA) and were cultured in serum-free DMEM for 6 days [24]. The cells were washed three times with DMEM and were incubated in DMEM containing 10, 20, 30, 50 and 100 ng/mL ActA for 24 h. The cells were washed three times with DMEM and were incubated for 6 h in DMEM containing 1 mmol/L NaS_2_O_4_ under hypoxic conditions (37 °C, 5% CO_2_ and 95% N_2_) in the absence of sugar [25].

### 4.3. Cell Viability Assay

The MTT method [26] was used to assess the cytotoxic effects of ActA. The cells were grown to a density of 5 × 10^4^ cells/well and were then treated with 10, 20, 30, 50 and 100 ng/mL ActA in a 96-well plate for 24 h. At the end of the treatment, the ActA-containing medium was carefully removed and the cells were treated with OGD for 6 h. The culture medium was removed and 200 μL medium containing MTT (20 μL, 5 mg/mL in PBS, Sigma, St. Louis, MO, USA) was added to each well. After 4 h of incubation at 37 °C, the medium was removed and DMSO (100 μL) was added to each well. The optical absorbance (A) of each well was read at 490 nm. The percentage of viable cells was calculated as follows: (A of experimental group/A of control group) × 100% [27].

### 4.4. Flow Cytometry Using Annexin V/PI Staining

For the quantitative assessment of apoptosis, Annexin V-FITC and PI double staining, followed by flow cytometry was used. The cells were detached using 0.25% trypsin and harvested, the cells were washed with cold PBS (4 °C) three times and floated by 300 μL Binding Buffer (1×). The cells were stained by 5 μL Annexin V-FITC (Kaiji Bio Co., Nanjing, China) for 15 min at room temperature in the dark and stained by 5 μL PI staining (Kaiji Bio Co., Nanjing, China) for 5 min. Cells were analyzed immediately using flow cytometry. The signals from apoptotic cells are localized in the lower right quadrant of the resulting dot-plot graph [28].

### 4.5. Western Blotting Analysis

After treatment with ActA and OGD for 6 h, the cells were washed twice using cold PBS and 1 × 10^6^ cells were lysed using RIPA buffer [50 mmol/L Tris (pH 8.0), 150 mmol/L NaCl, 0.1% SDS, 1% NP40 and 0.5% sodium deoxycholate] containing protease inhibitors (1% cocktail and 1 mmol/L PMSF) [29,30]. Total proteins were separated using 15% SDS-PAGE and were transferred to a PVDF membrane. The membrane was blocked using Tris-buffered saline with 0.1% Tween 20 (pH 7.6, TBST) for 1 h at room temperature and was incubated with the primary antibody solution (1:1,000) at 4 °C overnight. After two washes in TBST, the membrane was incubated with the HRP-labeled secondary antibody (Santa SC-2073) for 1 h at room temperature and was washed three times with TBST. The final detection was performed using enhanced chemiluminescence (ECL) Western blotting reagents (Amersham Biosciences, Piscataway, NJ, USA) and the membrane was exposed to Lumi-Film Chemiluminescent Detection Film (Roche). Loading differences were normalized using a monoclonal β-actin antibody. The antibodies used in the study included ActRIIA (Santa, mouse, SC-57022), Smad4 (Santa, rabbit, SC-73040), Smad3 (Santa, rabbit, SC-101154), caspase-3 (Santa, mouse, SC-7272), SOD (Santa, rabbit, SC-18503), NOS (Santa, mouse, SC-49055) and β-actin (Santa, mouse, SC-2021).

### 4.6. Caspase-3 Activation Assays

Caspase-3 colorimetric assay kits (Kaiji Bio Co., Nanjing, China) were used to investigate caspase-3 in PC12 cells after OGD and ActA treatment, according to the manufacturer’s instructions. Briefly, cells were lysed by incubation with cell-lyse buffer on ice for 1 h, and then centrifuged at 10,000 g for 1 min. Enzymatic reactions were carried out in a 96-well microplate. For each reaction sample, cell lysate (50 μL) was incubated with substrate for 4 h at 37 °C before measurement of the absorbance at 405 nm. Two additional controls, one without cell lysate and the other without substrate were included. Total protein was determined by the Coomassie Brilliant Blue method.

### 4.7. Measurement of NO

Nitrite production, measured by the Griess reaction, was used as a measure of NO production. Briefly, culture supernatant (100 μL) was incubated with an equal volume of Griess reagent (1 part 0.1% naphthylethylenediamine, 1 part 1% sulfanilamide in 5% H_3_PO_4_) in 96-well tissue culture plates for 10 min at room temperature in the dark [31]. The absorbance at 540 nm was determined using a microplate reader (SpectraMAX 340, Molecular Devices, Sunnyvale, CA, USA). The concentration of Nitrite was calculated as follows: 


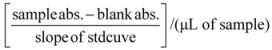


### 4.8. Measurement of SOD

Cells (1 × 10^5^) were harvested and after treatment with OGD for 6 h and ActA plus OGD for 6 h, the cells were washed twice with cold PBS and were lysed in ice-cold 0.1 M Tris/HCl (pH 7.4 containing 0.5% Triton X-100, 5 mM β-ME, 0.1 mg/mL PMSF). The cell lysates were centrifuged at 14,000 g for 5 min at 4 °C and the cell debris was discarded [32,33]. The total SOD activity in the supernatants (contain the cytosolic and mitochondrial fractions) were measured using the Superoxide Dismutase (SOD) Activity Assay Kit (Biovision k335-100, San Francisco, CA, USA) in accordance with the manufacturer’s instructions. The percentage of SOD was calculated as follows:


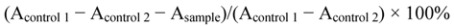


### 4.9. Statistics

All data were presented as mean ± S.E.M., based on data derived from three to six independent experiments. The square χ^2^ analysis was performed to evaluate the significance of inter-group differences. Student’s *t-*test was used for single comparison between two groups. Two-way ANOVA using the Student-Newman-Keuls method was adopted for comparison of variables after treatment. *p* < 0.05 was considered significant. All statistical calculations were performed using SigmaStat statistical software package (SPSS10.0, Chicago, IL, USA).

## 5. Conclusions

In conclusion, our results demonstrate that ActA protects PC12 cells in an OGD-deprivation model through the activation of the Smad pathway, the inhibition of NO production and the increased activation of SOD. Further investigation is required to understand fully the beneficial role of ActA in ischemic injury and could eventually lead to clinical interventions that will salvage brain cells that are at risk in ischemic cerebrovascular disease.
